# Effect of population stratification analysis on false-positive rates for common and rare variants

**DOI:** 10.1186/1753-6561-5-S9-S116

**Published:** 2011-11-29

**Authors:** Hua He, Xue Zhang, Lili Ding, Tesfaye M Baye, Brad G Kurowski, Lisa J Martin

**Affiliations:** 1 Division of Biostatistics and Epidemiology, Cincinnati Children’s Hospital Medical Center, Cincinnati, OH 45229, USA; 2 Division of Asthma Research, Cincinnati Children’s Hospital Medical Center, Cincinnati, OH 45229, USA; 3 Department of Pediatrics, University of Cincinnati School of Medicine, Cincinnati, OH 45267, USA; 4 Physical Medicine and Rehabilitation, Cincinnati Children’s Hospital Medical Center, Cincinnati, OH 45229, USA; 5 Division of Human Genetics, Cincinnati Children’s Hospital Medical Center, Cincinnati, OH 45229, USA

## Abstract

Principal components analysis (PCA) has been successfully used to correct for population stratification in genome-wide association studies of common variants. However, rare variants also have a role in common disease etiology. Whether PCA successfully controls population stratification for rare variants has not been addressed. Thus we evaluate the effect of population stratification analysis on false-positive rates for common and rare variants at the single-nucleotide polymorphism (SNP) and gene level. We use the simulation data from Genetic Analysis Workshop 17 and compare false-positive rates with and without PCA at the SNP and gene level. We found that SNPs’ minor allele frequency (MAF) influenced the ability of PCA to effectively control false discovery. Specifically, PCA reduced false-positive rates more effectively in common SNPs (MAF > 0.05) than in rare SNPs (MAF < 0.01). Furthermore, at the gene level, although false-positive rates were reduced, power to detect true associations was also reduced using PCA. Taken together, these results suggest that sequence-level data should be interpreted with caution, because extremely rare SNPs may exhibit sporadic association that is not controlled using PCA.

## Background

Genome-wide association studies (GWAS) have proved to be successful in identifying common single-nucleotide polymorphisms (SNPs) associated with complex and common traits [1,2]. One of the common problems in population-based GWAS is population stratification. Several approaches have been used to correct population stratification, including genomic control, structured association, and principal components analysis (PCA) [3,4]. PCA is used to infer axes of genetic variation that can be interpreted as describing continuous ancestral heterogeneity within a group of individuals [5]. Its effectiveness has been shown on common variants [3,6,7]. However, recent studies have demonstrated the importance of multiple rare variants in the etiology of complex diseases [8-10]. It is not clear whether PCA works on rare variants. Thus our purpose in this paper is to evaluate the effect of PCA on false-positive rates for common and rare variants at the SNP and gene level.

## Methods

We conduct all analyses using the 200 replicates of the unrelated individuals data simulated for Genetic Analysis Workshop 17 (GAW17), with the knowledge of the underlying simulation model [11]. We focus on the normally distributed phenotype Q1. Age, Sex, and Smoke status are included as covariates. Because most causal variants discovered so far are functional, we focus on nonsynonymous SNPs in the current study. We define variants with minor allele frequency (MAF) less than 1% as rare, and those with a MAF larger than 5% as common. To determine the significance level, we apply a linkage-disequilibrium-adjusted Bonferroni correction using a mean linkage disequilibrium correlation of 0.138 among common SNPs.

We assess association of Q1 with a gene or SNP using linear regression. At the SNP level, association is analyzed with an additive model. At the gene level, we use three methods to collapse rare variants within a gene: indicator, proportion, and data-adaptive sum test methods. The indicator and data-adaptive sum test methods are described in the GAW17 background methods paper [12]. We simplify the data-adaptive sum test without doing permutation. The proportion method was previously described by Morris and Zeggini [2]. Briefly, let *n_i_* denote the number of rare variants successfully genotyped for subject *i*, and let *r_i_* be the number of these variants at which the variant carries at least one copy of the minor allele. We define a new variable *S_i_* = *r_i_*/*n_i_*, the proportion of loci within a gene at which a subject carries a minor allele.

**Figure 1:**
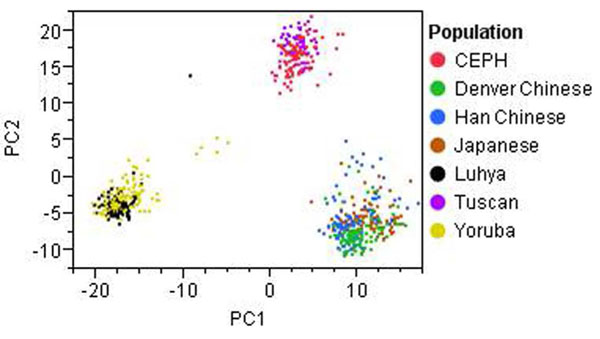
Scatterplot of the first two principal components

The GAW17 unrelated individuals data are divided into seven populations (CEPH [European-descended residents of Utah], Denver Chinese, Han Chinese, Japanese, Luhya, Tuscan, Yoruba) and thus may be susceptible to a form of confounding known as population stratification if the SNP or gene shows marked variation in allele frequency across subpopulations and if these subgroups also differ in their baseline risk of the disease [13]. To account for population stratification, we perform PCA using 1,379 common nonsynonymous SNPs (MAF > 0.05) to infer continuous axes of genetic variation. The first two principal components reveal clear distinctions among the three human ancestral origins (European, Asian, and African), accounting for 10.4% and 6.6% of the total variation, respectively (Figure 1). We use the first three principal components as covariates to adjust for population stratification based on the scree plot.

## Results

**Figure 2:**
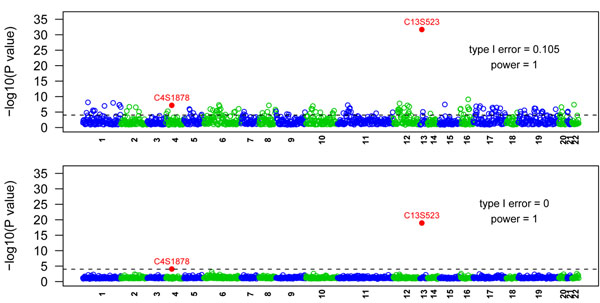
**Manhattan plot of 1,379 common nonsynonymous SNPs (MAF > 0.05).** Top panel: before PCA adjustment. Bottom panel: after PCA adjustment. Dashed line corresponds to the linkage-disequilibrium-adjusted Bonferroni significance level of 9.8 × 10^−5^.

**Figure 3:**
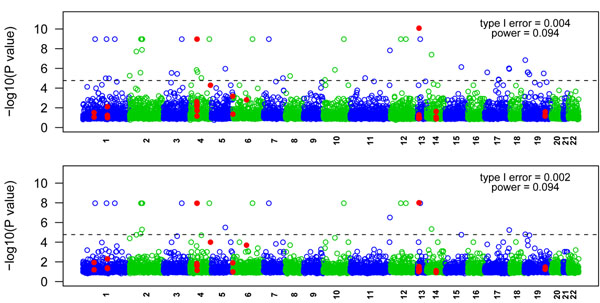
**Manhattan plot of 10,648 rare nonsynonymous SNPs (MAF < 0.01)**. Top panel: before PCA adjustment. Bottom panel: after PCA adjustment. Dashed line corresponds to the linkage-disequilibrium-adjusted Bonferroni significance level of 1.7 × 10^−5^.

Figure 2 shows the results of the single-SNP analysis for 1,379 common SNPs with and without PCA. We use the 95% quantile of the 200 *p*-values to represent the overall results of the 200 replicates. In the simulation, Q1 is influenced by 39 SNPs in 9 genes, including 2 common SNPs (MAF > 0.05) and 32 rare SNPs (MAF < 0.01). Our analysis detected the two causal common SNPs before and after population stratification adjustment. C13S523 has a relatively high MAF (0.165) with mild effect, and C4S1878 has a lower MAF (0.067) with moderate effect. In the analysis without PCA, 144 null SNPs were declared significant, leading to a false-positive rate (type I error) of 144/1,377 = 0.105. The false-positive rate dropped to 0 after adjusting for population stratification. Figure 3 is the Manhattan plot of 10,648 rare SNPs before and after PCA. Forty-four null SNPs were declared significant before PCA and 21 null SNPs were declared significant after PCA, leading to type I errors of 0.004 and 0.002, respectively.

**Figure 4:**
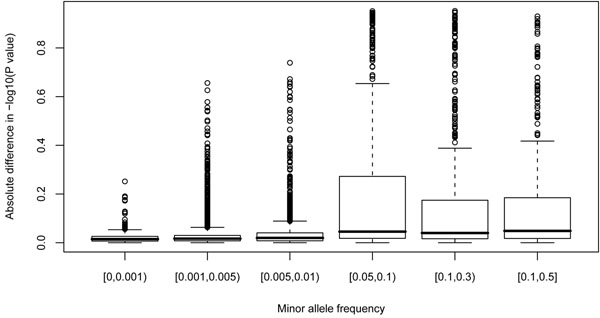
Boxplot of the absolute difference in −log10(*p*-value) before and after PCA by MAF

These results suggest a MAF-dependent effect of PCA. We next examined the absolute difference in −log_10_(*p*-value) before and after PCA for various MAFs (Figure 4). Our results show that the median difference increases with MAF. When comparing SNPs with low MAF (<0.01) with those with high MAF (>0.05), we detected statistical significance (Wilcox rank sum test, *p* < 2.2 × 10^−16^). These results suggest that principal components adjust the *p*-value more substantively for higher MAF SNPs.

**Figure 5:**
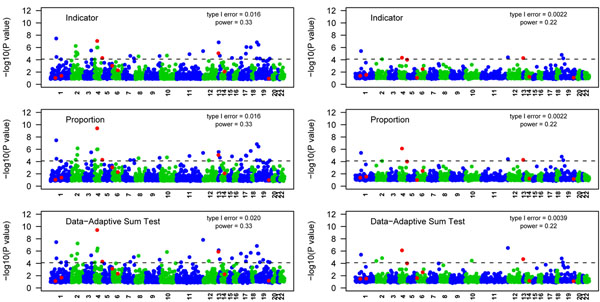
**Manhattan plot of genes for the three collapsing methods.** Left panels: before PCA adjustment. Right panels: after PCA adjustment. Dashed line corresponds to the linkage-disequilibrium-adjusted Bonferroni significance level of 7.86 × 10^−5^.

We also tested association at the gene level. We compared three collapsing methods before and after PCA (Figure 5). Before adjusting for population stratification, for all methods, three causal genes (*KDR*, *FLT1*, and *VEGFC*) were declared significant. Twenty-nine, 29, and 35 null genes were falsely detected for the indicator, proportion, and data-adaptive sum test methods, respectively (type I errors of 0.016, 0.016, and 0.020, respectively). After adjusting for population stratification, we detected two causal genes. The number of falsely detected genes was reduced dramatically to four, four, and seven, leading to type I errors of 0.0022, 0.0022, and 0.0039 for the indicator, proportion, and data-adaptive sum test methods, respectively. We then explored the effect of PCA on power. Table 1 describes the number of times each causal gene was detected across 200 simulations for the three methods. Overall, power to detect genes in individual replicates was low; only *KDR* was identified at greater than 80% power without PCA adjustment. Furthermore, with PCA adjustment, power dropped to about 25% for *KDR*. Comparing the three methods, we found that the indicator method had lower power to detect *KDR* with or without PCA adjustment. Adjustment for population stratification greatly reduced the number of false positives but also reduced the power to detect true genes.

**Table 1:** Number of replicates with true discovery for the causal genes before and after PCA adjustment

Gene	Indicator method		Proportion method		Adaptive-sum test method	
	
	Before PCA	After PCA	Before PCA	After PCA	Before PCA	After PCA
*ARNT*	0	0	0	0	0	0
*ELAVL4*	0	0	0	0	0	0
*FLT1*	33	12	33	12	67	15
*FLT4*	2	0	1	0	3	0
*HIF1A*	0	0	0	0	0	0
*HIF3A*	0	0	0	0	0	0
*KDR*	94	17	160	50	163	53
*VEGFA*	0	1	0	1	0	1
*VEGFC*	15	8	15	8	15	8

We also investigated the effect of population stratification on phenotypes Q2 and Q4 (data not shown). Q2 showed the same pattern as Q1, supporting our contention that PCA does not perform well for rare variants. Q4 is not associated with any SNPs and thus is used to assess the effect of PCA on false-positive rates. No significant association was identified before and after PCA. The effect of population stratification appeared to diminish.

## Discussion and conclusion

Using the GAW17 mini-exome data set, we have demonstrated that the MAF of SNPs influences the ability of PCA to effectively control false discovery. Specifically, PCA reduced false-positive rates more effectively in common SNPs than in rare SNPs. At the gene level, although false-positive rates were reduced, the power to detect true associations was also reduced using PCA.

Consistent with previous studies, PCA efficiently corrects for population stratification for common variants [3,6,7]. For rare variants, using principal components to adjust for population stratification also reduces the type I error but does not improve the power to detect causal variants. Importantly, we noticed that two causal rare SNPs (C4S1877 and C4S1889) were private SNPs and exhibited the mutant genotype in a single person (NA07347). For the other 14 nonsynomymous SNPs that exhibited strong association (C1S3619, C1S6350, C1S8205, C2S3362, C2S3482, C2S3613, C3S4002, C4S4650, C6S4373, C7S1247, C10S5614, C12S2922, C12S4373, and C13S768), the mutant form was also present only in individual NA07347. The mutant genotype is not very likely to distinguish the null SNPs from the two true causal SNPs because of identical genotype. Thus studies using sequence-level data should exhibit caution when interpreting the causality of extremely rare SNPs because these may be sporadic.

For the gene-level analysis, each method was underpowered to identify genes harboring rare causal variants, with none of the methods identifying more than 50% of the genes at a 50% success rate. All three methods had deflated type I error and low power. When comparing performance across the three methods, we found that the indicator method had the lowest power but that the data-adaptive sum test method was more susceptible to false-positive associations. These results suggest that PCA can be an effective method for reducing false positives in gene-level analyses, but there will be reduced power.

We applied PCA to genotype data to infer continuous axes of genetic variation. The principal components capture the continuous ancestral heterogeneity across subpopulations, which aligns well with common SNPs. But for rare SNPs, PCA does not correct for the sparse nature and sudden heterogeneity exhibited by rare variants. The linkage disequilibrium between rare variants is not as stable as the linkage disequilibrium between common SNPs, making it harder to adjust for population stratification. For rare variants as extreme as private SNPs, with the mutant genotype existing only in a single person, PCA using common variants may not be applicable to correct population stratification.

## Competing interests

The authors declare that there are no competing interests.

## Authors’ contributions

HH carried out the design of the study, performed the statistical analysis and drafted the manuscript. XZ participated in the discussion and helped to draft the manuscript. LD, TMB and BGK participated in the discussion and helped to edit the manuscript. LJM conceived of and oversaw the study, and helped to draft the manuscript. All authors read and approved the final manuscript.
